# Ordinal Conditional Entropy Displays Reveal Intrinsic Characteristics of the Rosenberg Self-Esteem Scale

**DOI:** 10.3390/e25091311

**Published:** 2023-09-08

**Authors:** Emanuela Furfaro, Fushing Hsieh

**Affiliations:** 1 Department of Statistics, University of Washington, Seattle, WA 98195, USA; 2 Department of Statistics, University of California, Davis, CA 95616, USA; fhsieh@ucdavis.edu

**Keywords:** ordinal categorical data, conditional Shannon entropy, mutual conditional entropy (MCE), network

## Abstract

Individual subjects’ ratings neither are metric nor have homogeneous meanings, consequently digital- labeled collections of subjects’ ratings are intrinsically ordinal and categorical. However, in these situations, the literature privileges the use of measures conceived for numerical data. In this paper, we discuss the exploratory theme of employing conditional entropy to measure degrees of uncertainty in responding to self-rating questions and that of displaying the computed entropies along the ordinal axis for visible pattern recognition. We apply this theme to the study of an online dataset, which contains responses to the Rosenberg Self-Esteem Scale. We report three major findings. First, at the fine scale level, the resultant multiple ordinal-display of response-vs-covariate entropy measures reveals that the subjects on both extreme labels (high self-esteem and low self-esteem) show distinct degrees of uncertainty. Secondly, at the global scale level, in responding to positively posed questions, the degree of uncertainty decreases for increasing levels of self-esteem, while, in responding to negative questions, the degree of uncertainty increases. Thirdly, such entropy-based computed patterns are preserved across age groups. We provide a set of tools developed in R that are ready to implement for the analysis of rating data and for exploring pattern-based knowledge in related research.

## 1. Introduction

Questionnaire items often require a single subject to make their choice of rating on a scale, for instance, of 1 to 4, with reference to a list of statements often aimed at studying individuals’ behaviors, attitudes and opinions. This type of data is ordinal, but methodologies introduced for treating numerical data are often used to analyze them [1,2,3]. However, while a single natural ordering exists in self-rating data, the distance between 1 and 2 is likely very different from the distance between 2 and 3, and so is for 3 and 4. The nature of self-rating scales is then far from being metric, which refers to measures based on a universal unit, such as the one given by the use of a ruler, that is valid at 1 as well as at 100.

From the perspective of two distinct subjects, say A and B, who answer the same self-rating question, it is known that A’s 1 is distinct from B’s 1. That is, A’s 1 could be B’s 2 or even 3. Therefore, there are two essential aspects that need to be considered when analyzing self-rating data. First, the comparability between different subjects’ rating scale is not certain. Secondly, A’s increasing scale: {1, 2, 3, 4}, can be transformed by an unknown order-preserving transformation to B’s increasing scale: {1, 2, 3, 4}. Since such an order preserving transformation is unknown, it is coherent and also conservative to treat A’s and B’s 1s as the same category. That is, we want to avoid resting our analysis on the fact that B’s 2 is larger than A’s 1. To extract meaningful information out of rating data, we only need to make our data analysis rest on the fact that ratings in category 1 are “collectively” smaller than ratings in category 2, and so on. It is worth emphasizing here that self-rating data are far from numerical. It is digital-coded for convenience. In other words, ‘1’, ‘2’, ‘3’ and ‘4’ are just labels, rather than numbers, and it would make no difference if the rating was alphabet-coded and ordered, such as {a, b, c, d}, or if it was another ordered set of numbers, such as {−100, −10, 1, 300}, as long as the respondent is informed that they are labels for ‘strongly disagree’, ‘disagree’, ‘agree’, ‘strongly agree’.

From the perspective of many distinct subjects, all person-specific self-rating scales, which do not contain metric information and possibly involve many unknown order-preserving transformations, are just too heterogeneous to allow sensible meaningful arithmetic operations, such as the calculation of mean, variance, correlation and the use of metric models. These operations could indeed become meaningless and may yield misleading conclusions [3]. Since ordinal data are common in many domains including psychology, medicine, economics, etc., it is relevant to expand the literature on the use of measures that respect data’s categorical nature and simultaneously provide simple and intuitive tools to explore patterns along the data’s ordinal axis.

The most robust approach for accommodating self-rating data is to make use of its most fundamental nature: being necessarily treated as categorical but displayed in an ordinal fashion. This is the intrinsic theme underlying the self-rating data. In fact, being categorical only allows for a grouping operation, and displaying in an ordinal fashion also accommodates the grouping operation. Grouping the same ratings from all subjects together is indeed the least unnatural and non-artificial operation, and displaying such groups in order is the least intrusive summary of self-rating data.

Drawing on the above points, in this paper, we show how the concept of conditional entropy, which is commonly used in Information Theory, is a suitable one when dealing with self-rating data. We also provide tools, developed in the R Statistical Software [4], which easily allow us to graphically explore patterns in self-rating data. In this paper, we use them to discuss “Self-esteem across gender and age” as a topic of human complex system within a research area where behavioral science, psychology and sociology intersect.

Prof. Morris Rosenberg studied dynamic changes of late adolescents’ self-image in his well-known 1965 book [5]. Boys and girls of 15 to 18 years of age are so-called late adolescents. This age group is marked by the urgent necessity of making drastic self-image changes in response to drastic physiological and psychological developments. Such developments are driven by changes in their own bodies as well as to the different societal potentials. The scientific question to Rosenberg was to define high self-esteem and lower self-esteem late adolescents. However, there are no precise definitions of high or low self-esteem on one hand, and there exists no universal criterion to classify who belongs to this category against other categories on the other hand. It is well recognized that a person’s self-esteem has a spectrum in the sense that even subjects belonging to the same category could still have heterogeneous behaviors in responding to the same situation under the same circumstances. In order to address his scientific question, Rosenberg designed his famous Self-Esteem Scale with 10 questions. Each one of 10 questions simulates a positive or negative situation-circumstance. The four self-rating categories allow the expression of heterogeneous degrees. Each question simultaneously plays two roles: (1) self-rating to a defined situation-circumstance; (2) self-declaring a person’s ordinal locality of self-esteem within the self-esteem spectrum.

For the second part, in the context of nowadays on-line surveys, a large proportion of subjects, who take part in such Rosenberg Self-Esteem Scale, are far beyond Rosenberg’s original domain of late adolescents. In fact, many subjects of such an on-line survey are far older. Whether the designed questionnaires fit all age groups is certainly a legitimate scientific question, which is beyond the scope of this paper. In this paper, we assume that all ten questions remain to play the double roles within a specific age group, and we also partition each age group into two subgroups with respect to gender. By doing so, we hope to compute the validity of Rosenberg’s original design within each gender-specific age group, to which we try to address the original scientific question, respectively, and then to reveal the effect of age and gender by comparing computed patterns across age and gender subgroups.

## 2. Nature of Conditional Shannon Entropy

For each question $Q_{i}$, a subject is asked to report a rating, for example, on a scale of 1 to 4. Therefore, there are four groups of individuals with respect to four possible outcomes: 1, 2, 3 and 4. Each group merely reflects the collection of subjects who gave the same rating to the same question. Hence, it is necessary to recognize that the digital group-ID is not numerical in any realistic sense. The group size collectively conveys the proportions of response-categories in all subjects. This proportion reveals one aspect of subject-composition from strongly disagreeing to strongly agreeing with the question concerned. The evenness-vs-unevenness pertaining to such a proportion-vector can be evaluated by Shannon entropy [6,7].

Any pairwise linkage between two questions, for instance $Q_{i}$ and $Q_{j}$, indicates their existential association. Such a relation implies that results of $Q_{i}$ can be used to predict results of $Q_{j}$ to some extent and vice versa. Such a predictive implication is also applied for a triplet of linked questions, say $Q_{i}$ being linked to $Q_{j}$ and $Q_{s}$, or $Q_{i}$ linked to $Q_{j}$ and $Q_{j}$ linked to $Q_{s}$ in a serial fashion. It may also be of interest to know whether results of $Q_{j}$ could be better predicted by bivariate results of $\left(Q_{i},Q_{s}\right)$. In this paper, we explore such predictive relations by having $Q_{j}$ as a response variable, while $Q_{i}$ or $\left(Q_{i},Q_{s}\right)$ are covariate features.

For two questions $Q_{i}$ vs $Q_{j}$ (row-vs-column) with, for instance, the usual 1, 2, 3 and 4 levels, the corresponding 4×4 group-partitions become a contingency table (Table 1). If we fix $Q_{j}$ as the response/dependent variable and $Q_{i}$ as the covariate variable, we can study the entropy of $Q_{j}$ conveyed by $Q_{i}$. The lower the entropy, the more information $Q_{i}$ conveys on $Q_{j}$.

The *k*-th row of the contingency table refers to the observations where $Q_{i}=k$, with k=1,…,4. It reveals the proportion vector of group-ID of $Q_{j}$ conditioning on all subjects who gave *k* as their rating for $Q_{i}$. The conditional entropy thus evaluates the evenness-vs-unevenness on the $Q_{j}$’s four categories, given that $Q_{i}=k$. With reference to Table 1, when $Q_{i}=1$, $Q_{j}$ is extremely more likely to be in category 4 rather than in 1, 2 and 3, since the proportion of units in 4 is much higher than that in 1, 2 and 3. If we, instead, look at $Q_{i}=4$, respondents seem more evenly spread across the first 3 categories, suggesting more uncertainty around the value of $Q_{j}$. Therefore, we can say that the uncertainty around $Q_{j}$ is reduced when $Q_{i}=1$ as compared to $Q_{i}=4$. The meaning of this conditional entropy would be more proper when it is rescaled with respect to the marginal entropy of $Q_{j}$. Notice that working with the four conditional entropies of $Q_{j}$ given $Q_{i}=$ 1, 2, 3 and 4 convey more information than their weighted version because of the ordinal nature of the values of $Q_{i}=$ 1, 2, 3 and 4.

It is worth noting that the predictive relation can be established with $\left(Q_{j},Q_{s}\right)$ as a bivariate response variable and $Q_{i}$ as a covariate variable since we can fuse two variables into one through their contingency table. Seemingly, we can consider two covariate variables $\left(Q_{i},Q_{s}\right)$ and use them to predict $Q_{j}$.

### Computing Conditional Entropy and Its Representation

In this section, we present the computational aspects of conditional entropy. In order to study patterns of entropy of a response item $Q_{j}$, from now on *Y* to follow the common notation for a response variable, by levels of a covariate $Q_{i}$, from now on *X* to follow the common notation for a covariate, we compute the row-wise conditional entropy as follows:

(1) $$H\left(Y\right|X=x)=-\sum_{y\in S_{Y}}\frac{p(x,y)}{p(x)}log\left(\frac{p(x,y)}{p(x)}\right),$$

where $S_{Y}$ is the set of possible values of *Y*, p(x) is the rows proportion, i.e., the estimate of the marginal probability of *X*, and p(x,y) is joint proportion, i.e., the joint probability of X,Y.

The conditional entropy of *Y* given *X* is then defined as the weighted sum of H(Y|X=x) for each possible value of *X*, using p(x) as the weights:

(2) $$H\left(Y\right|X)=-\sum_{x\in S_{X}}p\left(x\right)H\left(Y\right|X=x),$$

where $S_{X}$ is the set of possible values of *X*, and p(x) is the rows proportion, i.e., the vector of the estimated marginal probabilities of *X*.

Last, the Shannon’s entropy of *Y* is calculated as

(3) $$H\left(Y\right)=-\sum_{y\in S_{Y}}p\left(y\right)log\left(p\left(y\right)\right)$$

where p(y) is the column proportion, i.e., the vector of the estimated marginal probabilities of *Y*.

We then compare the amount of entropy conveyed by the covariate with the entropy of *Y* by re-scaling the conditional entropy by the unconditional entropy $\frac{H(Y|X)}{H(Y)}$.

A general measure of the strength of the association between two variables *X* and *Y* is provided by the mutual conditional entropy (MCE). This is defined as the average between $\frac{H(Y|X)}{H(Y)}$ and $\frac{H(X|Y)}{H(X)}$, and it can be seen as an alternative to traditional measures, which include mainly measures based on the chi-square statistic [8,9,10]. Using the Rosenberg Self-Esteem dataset, we will show how consistently using conditional entropy measures throughout our study provides useful insights that would not be revieled otherwise.

In order to evaluate whether the observed values of entropy are different between two groups, one may want to calculate confidence intervals. We consider building confidence intervals as follows. We fix the row totals and we consider the row-wise conditional proportions as an estimate of the conditional probabilities. The row total and the vector of estimated conditional probabilities will constitute the parameters of a multinomial distribution. We then generate *M* samples from a multinomial with those parameters, thus creating *M* matrices. Based on the *M* matrices, we can calculate *M* values of any measure of entropy described above. The *M* values of, for instance, row-wise conditional entropy will constitute a sample from its empirical distribution, and we use the empirical distribution to calculate confidence intervals.

Once an entropy measure has been calculated, a crucial step in summarizing the results is displaying them in a simple and coherent manner. In order to display row-wise conditional entropy, we use the ggplot2 library [11] in R, while in order to display mutual associations, we use tools from the R library igraph [12].

To allow the reproducibility of this study and to expand the availability of tools to discover patterns in categorical data, we have developed a set of R functions, available on Github at https://github.com/emanuelaf/ceda-rosenberg (accessed on 23 July 2023). The code includes the implementation of the above-mentioned formulas and confidence intervals, and it includes the code to reproduce the graphical representations in this paper, allowing researchers to use the same concepts and explore patterns of entropy in ordinal data. In fact, while there exist R libraries that implement some measures of entropy and information gain [13], to the best of our knowledge, there are no libraries in R that allow for the use of entropy-based measures to graphically and explicitly investigate patterns of entropy in categorical data.

The next section shows a real example application with the aim of illustrating how to use these concepts based on entropy to gain useful insights on a categorical set of data.

## 3. Data and Results

Thanks to the wide-spread accessibility of the Internet, the Rosenberg Self-Esteem Scale has become a popular on-line test. Persons, who are far outside the original domain of application of this test, have taken this test, and the responses were made available on Kaggle. The online platform has created a Self-Esteem Scale dataset, which can be found via the following link: https://www.kaggle.com/datasets/lucasgreenwell/rosenberg-self-esteem-scale-responses (accessed on 21 October 2021). This dataset contains the responses to the Rosenberg Self-Esteem Scale of 47,974 subjects, along with information on age, sex and country of residence.

Participants in the survey were asked to rate the following 10 items (from now on we will interchangeably use the term item or question) on a scale where 1 = strongly disagree, 2 = disagree, 3 = agree and 4 = strongly agree (0 = no answer):

- Q1. I feel that I am a person of worth, at least on an equal plane with others.
- Q2. I feel that I have a number of good qualities.
- Q3. All in all, I am inclined to feel that I am a failure.
- Q4. I am able to do things as well as most other people.
- Q5. I feel I do not have much to be proud of.
- Q6. I take a positive attitude toward myself.
- Q7. On the whole, I am satisfied with myself.
- Q8. I wish I could have more respect for myself.
- Q9. I certainly feel useless at times.
- Q10. At times I think I am no good at all.

Notice that the set of questions is composed of items which are positively posed, in the sense that answering 4 means high self-esteem (questions 1, 2, 4, 6, 7) and negatively posed items (questions 3, 5, 8, 9 and 10).

Among the respondents, the group of females is larger than that of males, with 29,182 and 17,801, respectively. The range of the respondents’ age is very wide, with respondents from age 10 to 70 years old. The first important question relates to the potential heterogeneity across the span of the gender-axis. A second reasonable question is to ask whether diverse systems are defined across the age-axis. These two are important issues when analyzing this Kaggle dataset of the Rosenberg Self-Esteem Scale. In this paper, we are guided more by the data analysis perspectives rather than by the perspectives of psychiatry, psychology and sociology. We want to shed some light on these two issues by discovering the data’s information content with emphasis on the data’s information heterogeneity.

Starting with the first question, we first focus on late adolescents, i.e., individuals between 15 to 18 years old for a total of 12,045 individuals. This is the largest age group and also an interesting one from a psychology perspective. In order to work on a more homogenous group, we further filter the data to consider respondents from the US only, that is, we consider 6435 individuals. The sample includes 4129 girls, 1977 boys, 135 adolescents who identified as ‘other’ and 16 who identified as ‘none’. Given the very small sample size of the categories ‘other’ and ‘none’, we remove those individuals, and thus work on a sample of 4129 individuals who identify as girls and live in the US and 1977 who identify as boys and live in the US.

### 3.1. Mutual Conditional Entropy: Differences between Males and Females

**Figure 1:**
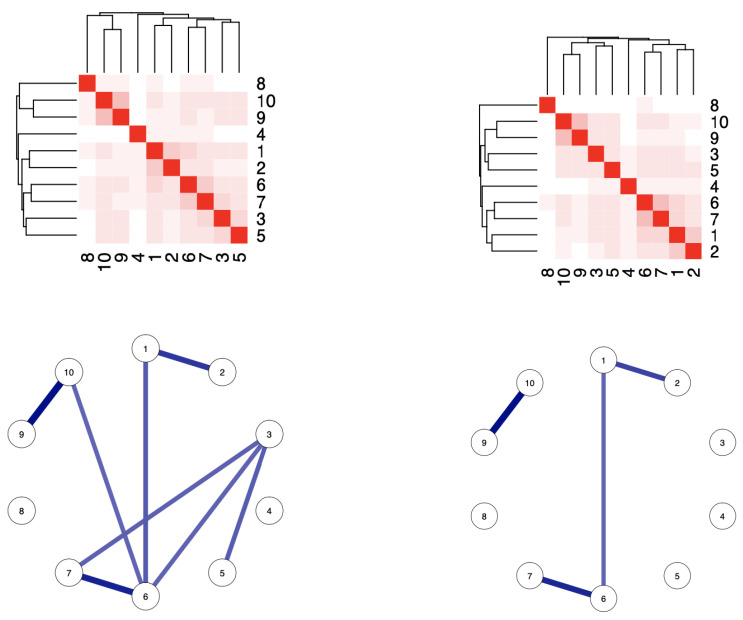
Males’ mutual conditional entropy (**left**) and females’ mutual conditional entropy (**right**). Numbers indicate the item and refer to the item number. Networks are built with linkages with a thickness proportional to 1- MCE and subject to a threshold of 0.2.

If we consider mutual conditional entropy as a measure of the strength of the association between items, we can obtain a general understanding of which items mutually convey more information. Figure 1 shows the heat maps and networks of male late adolescents’ and females’ mutual conditional entropy. Networks are built with linkages with a thickness proportional to 1- MCE, and, for the sake of readability, only values above 0.2 are displayed. The strongest associations are observed between items of the same “sign” and which are consecutive. This holds for both females and males; although, for the former, associations are consistently lower.

The network that refers to the sample of boys has more linkages between the 10 items, while the females’ network is more sparse. This is one important aspect of heterogeneity with respect to the gender axis. The four linkages of the females’ network are among clearly positive questions {1, 2, 6, 7} and strongly negative questions {9, 10}, respectively. In contrast, beyond the aforementioned four linkages, the males’ network distinctively contains 3 more linkages between clearly positive and clearly negative questions: {3, 6}, {3, 7} and {10, 6}. This network also reveals a linkage between two clearly, but not strongly, negative questions {3, 5}.

Such evident gender differences point to one conclusion that females to some evident degree are less certain (or more even) when facing non-strongly negative questions.

### 3.2. Row-Wise Conditional Entropy

Since calculating conditional entropy given a specific level of the covariate conveys different levels of uncertainty around the response, we now work on the row-wise conditional entropy. Row-wise conditional entropy means studying the uncertainty conveyed on a response variable by a specific group. Since groups correspond to people with high or low self-esteem (at least with respect to a specific question), studying row-wise conditional entropy may provide useful insights on the response patterns of people with high/low self-esteem.

For instance, let us consider $Q_{1}$ (I feel that I am a person of worth, at least on an equal plane with others) as the response and $Q_{7}$ (On the whole, I am satisfied with myself) as the covariate. The row-wise conditional entropy tells us whether those who answered ‘1’ to $Q_{7}$ (which are a group of people with low self-esteem) respond to answer $Q_{1}$ in a homogenous way and whether this is different from those who, for instance, answered ‘4’ to $Q_{7}$ (which correspond to a group of people with high self-esteem).

**Figure 2:**
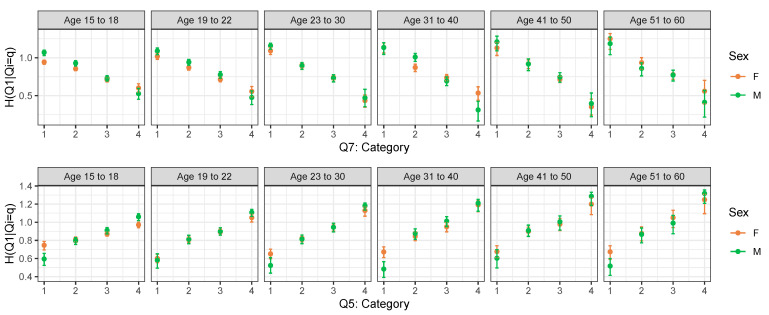
Row-wise conditional entropy of $Q_{1}$ given levels of $Q_{7}$ (top panel) and of $Q_{5}$ (bottom panel) by age group and sex.

From Figure 2, we can see that for increasing levels of $Q_{7}$, the entropy of $Q_{1}$ decreases. In other words, $Q_{1}$ is more concentrated and less spread out for higher levels of $Q_{7}$. Since high levels of $Q_{7}$ indicate a person with high self-esteem, it seems that these people convey with more certainty $Q_{1}$. This trend is consistent between gender and age groups, although the estimates are less precise as age increases due to the smaller sample size. Moreover, as age increases, the differences between the amount of information conveyed by high self-esteem people and low self-esteem people increases.

We now consider $Q_{5}$ as an explanatory variable. $Q_{5}$ is a negatively posed question; therefore, low levels of $Q_{5}$ indicate a person with high self-esteem. For increasing levels of $Q_{5}$, the entropy of $Q_{1}$ increases. In other words, $Q_{1}$ has a higher degree of uncertainty for higher levels of $Q_{5}$, i.e., for lower levels of self-esteem. These results confirm that self confident people answer with more certainty to $Q_{1}$.

**Figure 3:**
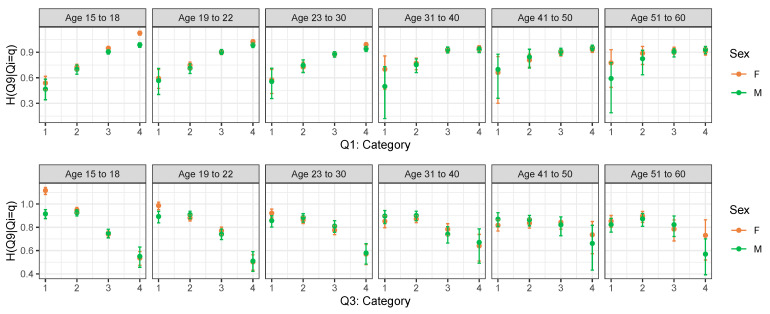
Row-wise conditional entropy of $Q_{9}$ given levels of $Q_{1}$ (top panel) and of $Q_{3}$ (bottom panel) by age group and sex.

A seemingly reverse but indeed “nonparallel” reasoning can be applied if we consider $Q_{9}$, which is a negatively posed question, as the response variable. From Figure 3, we can see that the entropy of $Q_{9}$ increases for increasing levels of a positively posed question and decreases for increasing levels of a negatively posed question. These results confirm that low self-esteem people answer with more certainty to $Q_{9}$.

**Figure 4:**
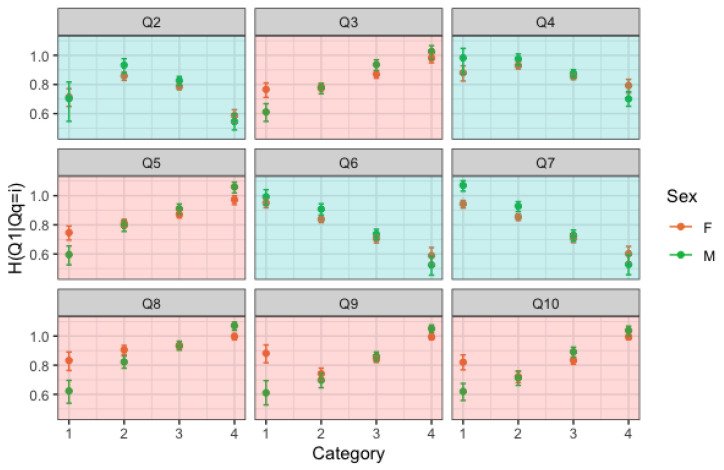
$H\left(Q_{1}\right|Q_{i}=k)$ and associated 95% confidence intervals by gender. Red plots refer to negatively posed questions, blue plots refer to positively posed questions.

We now consider only the largest age group, i.e., late adolescents. We fix the response variable as a positively posed question (for instance $Q_{1}$), and we let the covariate change. Looking at Figure 4, we can see that every time the covariate is a negatively posed question, a trend of decreasing entropy can be observed, while whenever we condition on levels of a negative item, then we observe a trend of increasing entropy. Note that the same trend, though not as clear, can be found in individuals of older ages, as previously described by Figure 2 and Figure 3.

**Figure 5:**
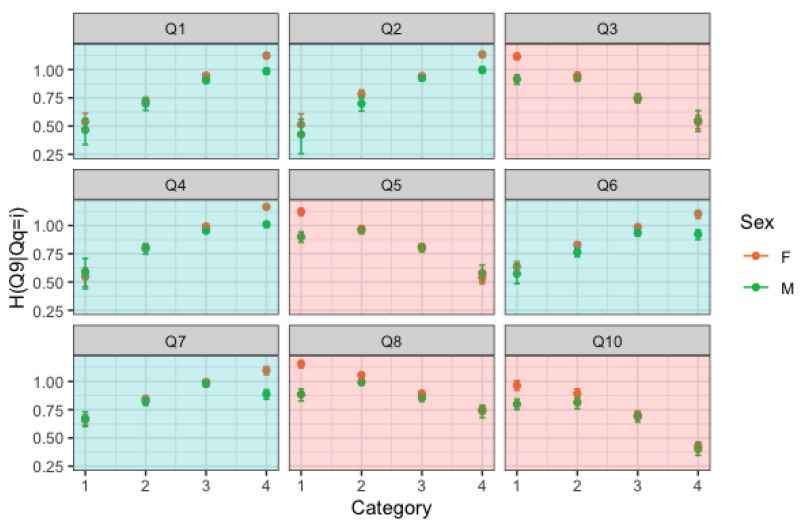
$H\left(Q_{9}\right|Q_{i}=k)$ and associated 95% confidence intervals by gender. Red plots refer to negatively posed questions, blue plots refer to positively posed questions.

The above observations are confirmed if we choose a negatively posed question as the response variable. Let us focus on the negatively posed covariates (red plots) of Figure 4 and on those who answered 1 in those questions. These represent groups of people with high self-esteem with regard to those items. There is a notable gender difference, with high self-esteem girls conveying more uncertainty than high-self esteem boys, which is fairly surprising and suggests some inconsistency. If we look at Figure 5, we again note that high self-esteem girls convey more uncertainty than high self-esteem boys.

Regarding the general trend, the results in Figure 4 are somewhat mirrored in Figure 5. In fact, the response variable is a negatively posed item, and there is increasing entropy if we condition on positive items and decreasing if we condition on a negative item.

### 3.3. Row-Wise Conditional Entropy by Gender: Two Covariates

Following the same idea, we now consider two variables as covariates. The conditioning variable is therefore now an item composed of 16 levels, 1-1, 1-2, 2-1, …, 4-3, 4-4.

**Figure 6:**
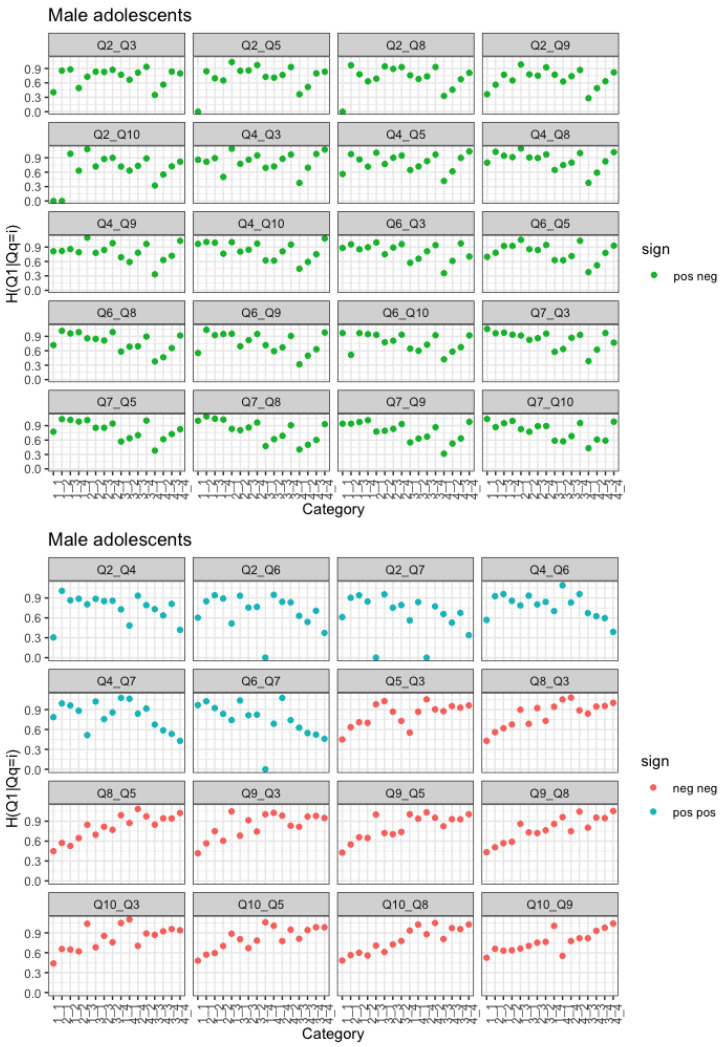
Row-wise conditional entropies for male adolescents. Bivariate conditioning variable and $Q_{1}$ as the response variable. Values of row-wise conditional entropy equal to zero correspond to those rows containing no observations.

**Figure 7:**
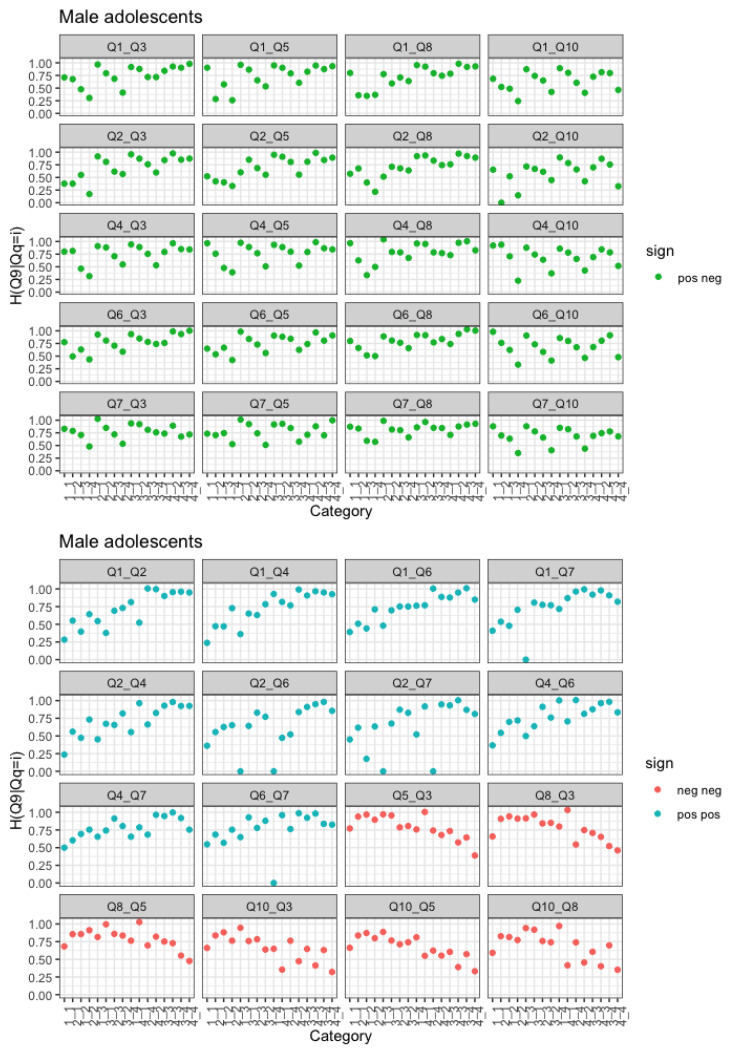
Row-wise conditional entropies for male adolescents. Bivariate conditioning variable and $Q_{9}$ as the response variable. Values of row-wise conditional entropy equal to zero correspond to those rows containing no observations.

If the response variable is a positive item and we condition on two positive items, the trend we observe is exactly as before, i.e., decreasing uncertainty with increased levels of self-esteem. If we condition on two negative items, again, the observed trend is the same as before, i.e., increasing uncertainty for decreasing levels of self-esteem. It is noted that the ordinal orders of the 16 levels on both positive or both negative questions can be clearly defined. However, it is not so in the case of one positive and one negative question. It is basically because the set of codes {1, 2, 3, 4} is categorical, not numerical. That is, negative questions {1, 2, 3, 4} are not necessarily equal to positive questions {4, 3, 2, 1}. If we condition on a combination of negative and positive items, a phenomenon similar to that of interactions can be observed. Looking at Figure 6, if we fix one of the levels of the positive item (i.e., the first one), we can see that entropy increases for increasing levels of the second item, which is a negative one. Of course, if we reversed the levels of one of the two items so that they would both go in the same direction, a reinforcing phenomenon, such as the one observed for items of the same sign, would be observed. Please note that while Figure 6 and Figure 7 refer to the sample of males, females exhibited a similar trend, and figures are available from the authors upon request.

## 4. Conclusions

The theme of this paper is to display a rating dataset’s information content in a way that is coherent with the data’s categorical and ordinal nature. Such a nature implies that metric-based statistical methodologies, such as correlation or principal component analysis (PCA) and several others, are not necessarily valid since arithmetical operations of mean and variance lose their meanings with respect to data’s categorical and ordinal nature. In sharp contrast, by summarizing rating data into contingency tables, the concept of conditional Shannon entropy naturally fits data’s categorical nature rather well, and the ordinal display of conditional entropy through a matrix lattice clearly provides a visible platform for pattern recognition and discoveries.

It is worth mentioning that the contingency table format importantly serves as the foundation for evaluating the reliability of any recognized patterns. Such reliability evaluations play a critical and fundamental role in interpreting data’s intrinsic information content, which is completely free of man-made structures and assumptions.

Our findings on the Rosenberg Self-Esteem Scale dataset are surprisingly clear. Patterns of conditional entropy with respect to the subject’s self-esteem axis ranging from strongly negative to strongly positive are rather reliably steady. In comparison, both age and gender factors have rather mild effects along the self-esteem axis. Nonetheless, the males’ associative network among the 10-question-nodes is more connected than females’ associative network. This global piece of information is saying that males’ ratings on the 10 questions tend to be less ambiguous than those of females.

Originally, this popularly used Rosenberg Self-Esteem Scale was conceptualized by its author as a single-factor scale, with scores ranging along a continuum of low self-esteem to high self-esteem [5]. This original intention seemingly is incoherent with our findings: people who, according to positively posed questions, have high self-esteem are more certain toward positive questions but more uncertain toward negative questions, while people who seem to have self-esteem when answering negatively posed questions, are more certain on negatively posed questions and more uncertain on positive ones. These two trends are neither exactly orthogonal, nor exactly parallel, but they do suggest that our findings are incoherent with Rosenberg’ original single-factor intent.

Further, there were three versions of rewording on the Rosenberg Self-Esteem Scale devised in [14]. With a much smaller dataset being collected and analyzed via Factor Analysis, [15], indeed, indicated that the original version fits a two-factor model, while positive- and negative-reworded versions fit single-factor models. It is noted that these results based on Factor Analysis might be fundamentally distinct with our patterns discovered under this study here.

The results found are easily reproducible, and researchers in the field of behavioral and social statistics who work with ordinal data can use a similar structure to explore patterns of conditional entropy with respect to the subject’s characteristics.

From an information content point of view, it is important to note that the most fundamental and essential information content of self-rating data is the collective patterns derived from all possible bivariate-questions, for instance, ($Q_{i}$, $Q_{j}$): if question $Q_{i}$ is taken as a response variable to be used for collecting subjects’ self-rating, then the question $Q_{j}$ is taken as a covariate to be used for collecting subjects’ self-declared status within the self-esteem spectrum. In other words, computing patterns pertaining to this bivariate-question ($Q_{i}$, $Q_{j}$) would be directional because of the inconsistency of subjects’ self-rating across the ten questions. For instance, a subject might give two different ratings to two relatively similar positive or negative questions.

From the collective perspective of the data’s information content, some bivariate-questions might contribute more than others since the ten questions are heterogeneous in their degrees of being positive and negative. Some computed patterns are clear for some bivariate-questions, but some are ambiguous. Thus, the true task facing a data analyst here is how to present and summarize potentially heterogeneous pattern information. In view of such computational and expositional tasks, we are confident that no model-based data analytics could work well in the sense of extracting authentic information content.

Beyond the bivariate-question, we considered a triplet-question ($Q_{i}$, $Q_{j}$, $Q_{k}$), with $Q_{i}$ being the response variable and ($Q_{j}$, $Q_{k}$)-pair being a 2D covariate variable. Theoretically speaking, such a triplet-question format will bring distinct information content from that derived from the bivariate-question. However, practical questions reside on how to make sensible ordinals among the 16 pairwise ratings. For this difficulty, we refrain from taking ($Q_{i}$, $Q_{j}$) as a bivariate response variable.

Last, it is worth noting that using entropy-based measures does not rely on assumptions of linear relation between items. Other measures are widely used in the literature to compute correlation of ordinal variables such as Spearman Rho, Kendall Tau or Polychoric [16,17,18,19]. In our dataset, these measures do provide similar results in cases where there is a linear relation between items (for instance, Q9 with Q10, Q1 with Q2, etc.). However, we would like to emphasize that the entropy approach we use is free from assumptions on the shape of the relationship.

## Figures and Tables

**Table 1:** Illustrative example from a subset of the Rosenberg dataset, late adolescents boys. $Q_{j}$ = ‘I feel that I am a person of worth, at least on an equal plane with others’, $Q_{i}$ = ‘At times I think I am no good at all.’ 1 = strongly disagree, 2 = disagree, 3 = agree and 4 = strongly agree.

	$Q_{j}$			
$Q_{i}$	1	2	3	4
1	11	13	110	337
2	2	22	220	153
3	20	135	328	122
4	117	186	157	44

## Data Availability

Not applicable.

## References

[1] Goodman L.A. (1978). Analyzing qualitative/categorical data. Log-linear Models and Latent Structure Analysis.

[2] Agresti A. (2003). Categorical Data Analysis.

[3] Liddell T.M., Kruschke J.K. (2018). Analyzing ordinal data with metric models: What could possibly go wrong?. J. Exp. Soc. Psychol..

[4] R Core Team (2021). R: A Language and Environment for Statistical Computing.

[5] Rosenberg M. (1965). Society and the Adolescent Self-Image.

[6] Shannon C.E. (1951). Prediction and Entropy of Printed English. Bell Syst. Tech. J..

[7] Cover T.M., Thomas J.A. (1991). Information theory and statistics. Elem. Inf. Theory.

[8] Kendall M.G. (1948). The Advanced Theory of Statistics.

[9] Yule G.U., Kendall M.G. (1950). An Introduction to the Theory of Statistics.

[10] Goodman L.A., Kruskal W.H. (1979). Measures of association for cross classifications. Measures of Association for Cross Classifications.

[11] Wickham H. (2016). Ggplot2: Elegant Graphics for Data Analysis.

[12] Csardi G., Nepusz T. (2006). The igraph software package for complex network research. InterJournal Complex Syst..

[13] Hausser J., Strimmerr K. (2021). Entropy: Estimation of Entropy, Mutual Information and Related Quantities. R Package Version 1.3.1. https://cran.r-project.org/web/packages/entropy/.

[14] Greenberger E., Chen C.-S., Dmitrieva J., Susan P., Farruggia S.P. (2003). Item-wording and the dimensionality of the Rosenberg Self-Esteem Scale: Do they matter?. Personal. Individ. Differ..

[15] Child D. (2006). The Essentials of Factor Analysis.

[16] Myers L., Sirois M.J. (2004). Spearman correlation coefficients, differences between. Encyclopedia of Statistical Sciences.

[17] Ghalibaf M.B. (2020). Relationship between Kendall’s tau Correlation and Mutual Information. Rev. Colomb. EstadíStica.

[18] Kiwanuka F., Kopra J., Sak-Dankosky N., Nanyonga R.C., Kvist T. (2022). Polychoric correlation with ordinal data in nursing research. Nurs. Res..

[19] Yu H., Hutson A.D. (2022). A robust Spearman correlation coefficient permutation test. Commun. Stat. Theory Methods.

